# T79. AFFECTIVE FACE PROCESSING IN SCHIZOPHRENIA: DISORDER-SPECIFIC OR TRANSDIAGNOSTIC DEFICIT?

**DOI:** 10.1093/schbul/sby016.355

**Published:** 2018-04-01

**Authors:** Jack Cotter, Kiri Granger, Jennifer Barnett

**Affiliations:** Cambridge Cognition

## Abstract

**Background:**

Social cognitive dysfunction is common in patients with schizophrenia and is associated with marked and persistent functional disability. Facial emotion recognition is a core aspect of social cognition and has been consistently demonstrated to be impaired in this population. However, it remains unclear whether these deficits are unique to patients with schizophrenia. We compared the severity of facial emotion recognition deficits in patients with both sub- and full-threshold psychotic symptoms to those observed across a range of psychiatric, neurological and developmental disorders in order to determine to what extent this represents a disorder-specific or transdiagnostic aspect of cognitive dysfunction.

**Methods:**

We conducted an electronic database search in order to identify published, peer-reviewed meta-analyses that compared facial emotion recognition task performance between individuals meeting clinical criteria for a psychiatric, neurological or developmental condition against healthy controls. Facial emotion recognition standardized mean difference effect size estimates (Cohen’s d or Hedges’ g) were required to have been derived from tasks in which participants had to identify, label or match images of faces consisting of all or any combination of the six basic emotions (happiness, sadness, anger, fear, surprise or disgust). Where possible, a ‘total’ score was used, comprising performance across multiple emotions. Effect size estimates must have been derived from two or more independent studies in order for the meta-analysis to be included. Where there were multiple publications for a given medical condition that met our inclusion criteria, we included the most recently published paper.

**Results:**

We identified 19 meta-analyses eligible for inclusion that examined performance across relevant tasks among 24 different clinical populations. Though the effect sizes are not directly comparable across clinical conditions (due to methodological differences between studies and in meta-analytic procedures), they demonstrate consistent and statistically significant deficits in facial emotion recognition across almost all of the clinical groups included in this review. Effect size estimates indicated that deficits among patients with schizophrenia were among the largest and most robust. Deficits were also evident even among those individuals with sub-threshold psychotic symptoms who met clinical criteria for being at ultra-high risk of developing a psychotic disorder.

**Discussion:**

Facial emotion recognition deficits are a transdiagnostic issue, potentially serving as a biomarker of neurological abnormality. However, these impairments appear to be particularly severe and debilitating among people with schizophrenia. There are currently no recognized treatments for these deficits. This in part is due to a lack of outcome measures suitable for use in clinical trials. Improved characterization and operationalization of social cognition and other ‘hot’ cognitive processes are necessary to facilitate and advance treatment efforts, both in schizophrenia and across other clinical groups. We are currently in the process of developing and acquiring normative data for a series of computerized tasks which can be used to assess these domains. This includes new variants of established tests which have been used to assess facial emotion recognition, as well as novel tasks to detect emotional biases and assess responses to socially-relevant information. These tasks will help to facilitate further research into these complex social processes and potentially assist in the development of interventions for those patients that are adversely affected.

